# Anti-Adipogenic Effect of Neferine in 3T3-L1 Cells and Primary White Adipocytes

**DOI:** 10.3390/nu12061858

**Published:** 2020-06-22

**Authors:** Miey Park, Jinyoung Han, Hae-Jeung Lee

**Affiliations:** 1 Department of Food and Nutrition, College of BioNano Technology, Gachon University, Gyeonggi-do 13120, Korea; mieyp@naver.com (M.P.); hanalice@gc.gachon.ac.kr (J.H.); 2 Institute for Aging and Clinical Nutrition Research, Gachon University, Gyeonggi-do 13120, Korea

**Keywords:** neferine, 3T3-L1 preadipocytes, differentiation, anti-adipogenic activity

## Abstract

Neferine, an alkaloid component extracted from lotus seed embryos, is known for its anti-inflammatory, anticancer, and antioxidant properties. However, the anti-adipogenic activity of neferine has not been thoroughly investigated. In this study, neferine was found to inhibit lipid accumulation in a dose-dependent manner during the differentiation of 3T3-L1 cells without inducing cytotoxicity. Real-time polymerase chain reaction and immunoblot analysis revealed the downregulation in the expression of peroxisome proliferator activated receptor gamma (PPARγ), CCAAT/enhancer-binding protein alpha (C/EBPα), sterol regulatory element-binding protein-1c (SREBP-1c), and fatty acid synthase (FAS) and the upregulation in carnitine palmitoyltransferase-1 (CPT-1) and sirtuin 1 (SIRT1) levels following neferine treatment. Furthermore, neferine increased the phosphorylation of adenosine monophosphate-activated protein kinase (AMPK) and acetyl-CoA carboxylase (ACC), which is an important regulator of fatty acid oxidation. Our result indicates that neferine attenuates adipogenesis and promotes lipid metabolism by activating AMPK-mediated signaling. Therefore, neferine may serve as a therapeutic candidate for obesity treatment.

## 1. Introduction

The prevalence of obesity, one of the biggest health problems among all age groups, is increasing worldwide [1,2]. In 2014, about 30% of the world’s population was estimated to be overweight or obese [3]. Obesity is characterized by the excessive accumulation of adipocytes, leading to a rise in body weight. It is a critical predictor of numerous comorbidities such as cardiovascular disease, insulin resistance-related diabetes, cancer, and depression [4,5,6]. Common weight loss cures in obese individuals include diets, physical activity, behavioral therapies, and pharmacological treatments [7]. Anti-obesity drugs involved in weight regulation are known to exert harmful side-effects, including headache and blood pressure abnormalities [8,9]. Hence, studies have been directed to investigate the potential role of plants to treat obesity and related metabolic disorders and to elucidate their beneficial effects on lipid and glucose metabolism [10].

Neferine is a bisbenzylisoquinoline alkaloid isolated from the seed embryo of *Nelumbo nucifera*, commonly known as lotus [11]. It has been consumed for a long time in India and China [12]. Neferine has been found to exhibit therapeutic properties such as antioxidant, anti-inflammatory, anticancer, and anti-amnesic effects [13,14,15]. Considering these beneficial properties, neferine may be exploited for the development of curative products with no side-effects [11]. For years, the embryos of *N. nucifera* seeds have been consumed in China and India to ameliorate various diseases and its typical bisbenzylisoquinoline alkaloid is neferine [11,12,16]. Previous studies have demonstrated its antioxidation, anti-inflammation, and anticancer properties [13,14,15]. Neferine can be potentially useful to treat cardiovascular diseases such as arrhythmia, thrombosis, and platelet aggregation [17,18]. Further, it is known to exert protective effects against Alzheimer’s disease, amnesia, and depression [15,19,20], which is suggestive of the plausible application of this phytochemical for curative purposes [18].

The differentiation of precursor cells into mature adipocytes is controlled by several markers associated with adipogenesis [21]. The transcription factors, peroxisome proliferator-activated receptor-γ (PPARγ), CCAAT/enhancer-binding proteins (CEBPs), and sterol regulatory element-binding proteins (SREBPs) are key regulators of adipogenesis [22,23,24], and AMP-activated protein kinase (AMPK) is a chief regulator of the underlying molecular mechanism [25]. Mitochondrial beta-oxidation plays an important role in energy metabolism and is regulated by carnitine palmitoyltransferase-1 (CPT-1) and acetyl-CoA carboxylase (ACC) [26]. AMPK upregulates the activity of CPT-1 and increases the transport of free fatty acids for beta-oxidation through the inhibition of the phosphorylation of ACC and decreases in the concentration of malonyl-CoA [25,27,28]. Further, it suppresses the expression of ACC and fatty acid synthase (FAS), which are critical transcription factors of lipogenesis, by inhibiting SREBP-1c activity [29]. Until now, very few studies have investigated the anti-adipogenic/lipogenic effect of neferine in 3T3-L1 preadipocytes. In the present study, we evaluate the effects of neferine on adipogenesis and lipid metabolism of 3T3-L1 preadipocytes.

## 2. Materials and Methods

### 2.1. Materials

Neferine (C38H44N2O6) was purchased from Sigma (St. Louis, MO, USA) and solvated in dimethyl sulfoxide. 3T3-L1 preadipocytes were acquired from the ATCC (Manassas, VA, USA). Cell growth medium (DMEM), bovine calf serum (BCS), trypsin, fetal bovine serum (FBS), and insulin were supplied by Thermo Fisher (San Jose, CA, USA), while antibiotic-antimycotic solution, 5-aminoimidazole-4-carboxamide ribonucleotide (AICAR), dorsomorphin (Compound C), 3-isobutyl-1-methylxanthine (IBMX), and dexamethasone (DEX) were procured from Sigma (St. Louis, MO, USA).

### 2.2. Cell Culture and Differentiation of Preadipocytes

3T3-L1 preadipocytes were grown in DMEM supplemented with 10% BCS and antibiotic-antimycotic solution in a 5% CO_2_ incubator at 37 °C. Differentiation of preadipocytes was induced by substituting the medium with DMEM containing 10% FBS and adipocyte differentiation cocktail (MDI; 1 μM DEX, 0.5 mM IBMX, and 10 μg/mL insulin) for 3 days.

C57BL/6 mice (Five-week-old males) were used for the isolation of primary adipocytes and stromal vascular fraction (SVF), as per the protocol described in the journal [30]. In brief, lumps of fat tissues collected from mice were minced with scissors and incubated with phosphate-buffered saline (PBS; Thermo Fisher, San Jose, CA, USA) supplemented with 1.5 U/mL of collagenase D (Sigma, St. Louis, MO, USA) at 37 °C for 30 min to 1 h. The lysate obtained was filtered with 40-mm cell strainers (SPL Life Science, Pocheon-si, Gyeonggi-do, Korea) and washed with PBS. After centrifugation (1200 rpm, 10 min) the cells were resuspended in DMEM. Adipocytes were cultured in SVF culture medium and incubated at 37 °C and 5% CO_2_ atmosphere. The primary white adipose tissue was subjected to differentiation using MDI, as previously described. Neferine was prepared at 20 mM and used to treat 3T3-L1 preadipocytes and SVF cells at 1, 2.5, 5, and 10 μM concentrations. The negative control was undifferentiated cells, while the positive control included differentiated cells without neferine treatment. 3T3-L1 preadipocytes were treated with an activator or inhibitor of AMPK. AICAR (10 μΜ) or dorsomorphin (5 μΜ) was added during differentiation until the cells were harvested. All experiments were carried out in triplicates, as per the guidelines for the care and use of laboratory animals of Gachon University (reference number: GIACUC-R2019004).

### 2.3. Cell Viability Assay

Preadipocytes 3T3-L1 cells were planted in 96-well plates (1 × 10^4^ cells/well) and allowed to adhere and grow for 24 h. Next, the cells were treated with neferine at 1, 2.5, 5, and 10 μM concentrations and incubated at 37 °C for 24, 48, and 72 h under 5% CO_2_ atmosphere. Cells were subjected to Cell Counting Kit-8 (Dojindo Molecular Technologies, Rockville, MD, USA) assay, as recommended by the manufacturer. The absorbance was measured at 450 nm using a microplate reader (BioTek Inc., Winooski, VT, USA).

### 2.4. Lipids Quantification

Experimental control, or neferine-treated 3T3-L1 cells, were rinsed and fixed using 4% paraformaldehyde for an hour or longer. Cells were gently washed with 60% isopropanol and allowed to dry. Each well was stained using a filtered Oil Red O working solution in isopropanol: distilled water for 1 h at room temperature (20–22 °C). Images of stained lipid droplets were obtained under an inverted microscope (Nikon Eclipse, Shinagawa, Tokyo, Japan). The dye was dissolved in 100% isopropanol, and the absorbance was read at 500 nm wavelength.

### 2.5. Quantification of Gene Expression

Total RNA was isolated by the TaKaRa^®^ method according to the instructions of the manufacturer (TaKaRa Bio, Kusatsu, Shiga, Japan). In total, 2 μg of isolated RNA was used for quantification using QuantStudio 3 (Thermo Fisher Scientific, San Jose, CA, USA), and 50 ng RNA was reversely transcribed to complementary DNA using a PCR (TaKaRa Bio, Kusatsu, Shiga, Japan). RT-PCR was performed using TB Green (TaKaRa Bio, Kusatsu, Shiga, Japan), and all reactions were carried out in triplicates. The sequences of forward and reverse primer sets are shown in Table 1.

### 2.6. Protein Quantification and Immunoblot Analysis

To analyze the expression of proteins such as PPARγ, C/EBPα, SREBP-1, FAS, CPT-1, AMPK, ACC, and β-actin, the cells treated with different concentrations of neferine were subjected to western blot analysis. 3T3-L1 adipocytes and primary white adipocytes were extracted with a protein lysis buffer (iNtRON Biotechnology, Seongnam, Korea) containing protease and phosphatase inhibitors (Thermo Fisher, San Jose, CA, USA). After incubation for 30 min on ice, total protein from each sample was quantified using a PRO-MEASURE protein measurement solution (iNtRON Biotechnology, Seongnam, Korea). Samples with same protein amounts were separated on SDS-PAGE gel, and the separated bands were electro-transferred to a polyvinylidene fluoride (PVDF) membrane. For 1 h, the membrane was blocked and immunoblotted with primary antibodies for 2 h, followed by probing with horseradish peroxidase-labeled secondary antibodies for 1 h. The reactive bands of target proteins were detected by the Quant LAS 500 system (GE Healthcare Bio-Sciences AB, Björkgatan, Uppsala, Sweden) using an enhanced chemiluminescence (ECL) reagent (Amersham Pharmacia, Little Chalfont, Buckinghamshire, UK).

### 2.7. Statistical Analysis

All experiments were independently performed in triplicates and presented as mean ± standard deviation (SD). Data were analyzed on GraphPad Prism 5.03 (GraphPad Software Inc., La Jolla, CA, USA) using the one-way analysis of variance (ANOVA) followed by Tukey’s post-hoc test. Probability (*p*) values less than 0.05 (*) were considered statistically significant.

## 3. Results

### 3.1. Effect of Neferine on the Viability of 3T3-L1 Cells

**Figure 1:**
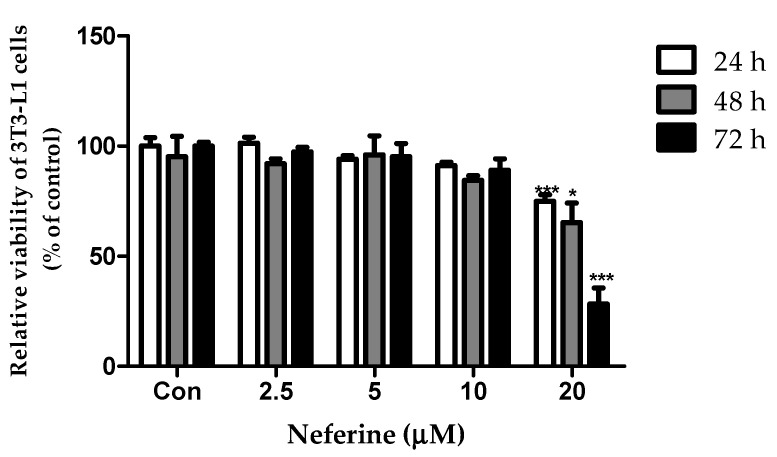
Effects of neferine on 3T3-L1 preadipocyte viability. Neferine was used at 2.5, 5, 10, and 20 μM concentrations for 24, 48, and 72 h. * *p* < 0.05 and *** *p* < 0.001 vs. Con. All data are presented as mean ± SD, and experiments were performed for at least three times. The positive control (Con) was differentiated 3T3-L1 cells treated with adipocyte differentiation cocktail.

A cell viability assay was used to investigate the cytotoxicity of neferine on 3T3-L1 preadipocytes. At 20 μM concentration, neferine significantly reduced the viability of cells after treatment for 24 and 72 h (Figure 1). Therefore, 10 μM neferine concentration was used in subsequent experiments.

### 3.2. Effect of Neferine on Intracellular Lipid Accumulation in 3T3-L1 Adipocytes

**Figure 2:**
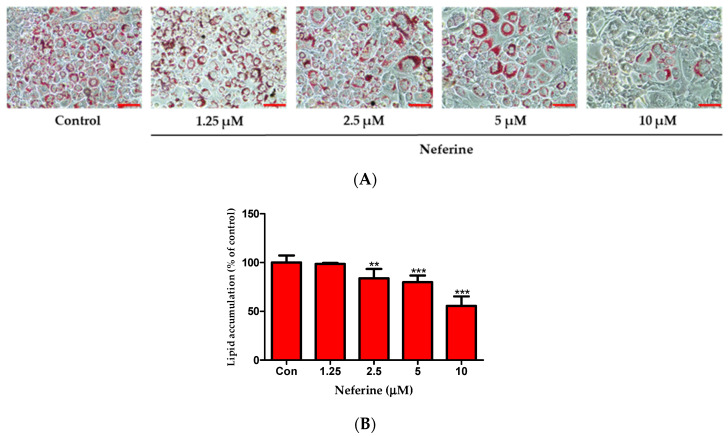
Effects of neferine on intracellular lipid accumulation. (**A**) Lipid droplets were measured by Oil Red O staining. Cell were treated with neferine at concentrations of 1, 2.5, 5, and 10 μM. Scale bar indicates 100 µm. (**B**) Relative lipid content is expressed as percentage. ** *p* < 0.01 and *** *p* < 0.001 vs. Con. All data are presented as mean ± SD, and experiments were performed at least thrice. The positive control (Con) was differentiated 3T3-L1 cells treated with adipocyte differentiation cocktail.

After inducing differentiation for 7 days, 3T3-L1 preadipocytes were stained with Oil Red O dye to observe intracellular lipid accumulation (Figure 2A). In comparison with control cells, those treated with neferine (1.25, 2.5, 5, and 10 μM) showed a significant decrease in lipid content in a dose-dependent manner (Figure 2B). These results demonstrated that neferine was involved in the inhibition of 3T3-L1 cell differentiation and lipid accumulation.

### 3.3. Effect of Neferine on the Adipogenesis of 3T3-L1 Cells

**Figure 3:**
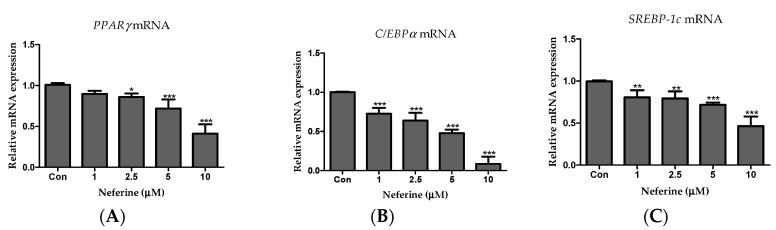
Effects of neferine on the expression of adipogenic marker genes. PCR was used to assess mRNA expression levels of (**A**) PPARγ, (**B**) C/EBPα, and (**C**) *SREBP1c*. * *p* < 0.05, ** *p* < 0.01, and *** *p* < 0.001 vs. Con. All data are presented as mean ± SD, and experiments were performed at least thrice. The positive control (Con) was differentiated 3T3-L1 cells treated with adipocyte differentiation cocktail.

To investigate the effects of neferine on adipogenesis, we performed RT-PCR. As shown in Figure 3, neferine significantly decreased the mRNA expression levels of the key adipogenic transcription factors, PPARγ, C/EBPα, and SREBP-1c (Figure 3A–C).

**Figure 4:**
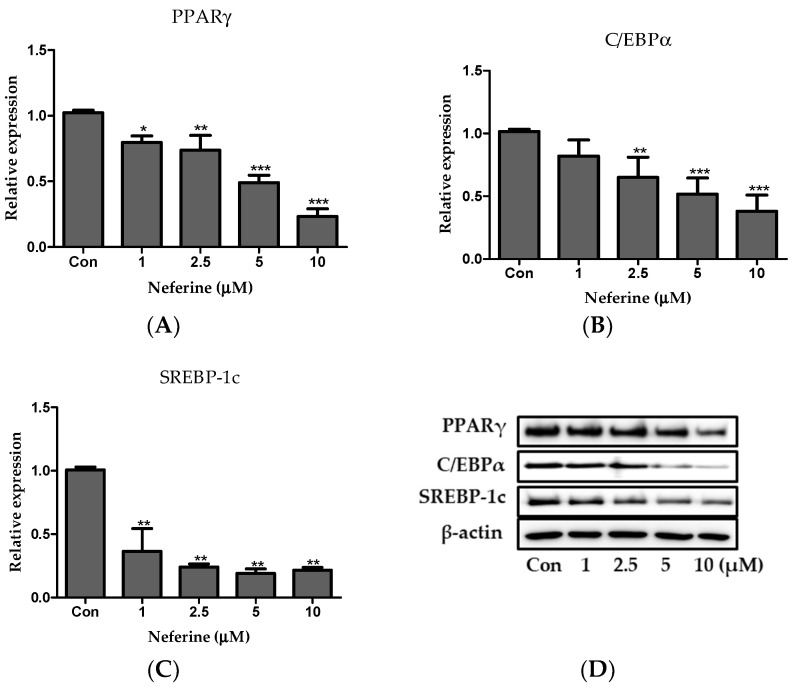
Effect of neferine on adipogenesis. Protein expression levels of (**A**) PPARγ, (**B**) C/EBPα, and (**C**) SREBP-1c were analyzed by immunoblotting. (**D**) Immunoblot results of adipogenic factors in 3T3-L1 cells. These results are expressed following normalization with β-actin level. * *p* < 0.05, ** *p* < 0.01, and *** *p* < 0.001 vs. Con. All data are presented as mean ± SD, and experiments were performed at least thrice. The control (Con) was positive control that differentiated 3T3-L1 cells treated with adipocyte differentiation cocktail.

The relative protein levels of PPARγ, C/EBPα, and SREBP1c in neferine-treated cells reduced in a dose-dependent manner (Figure 4A–D). Taken together, these data indicated that neferine downregulated the expression of the key factors associated with adipogenesis.

### 3.4. Effect of Neferine on Fatty Acid Oxidation in 3T3-L1 Adipocytes

**Figure 5:**
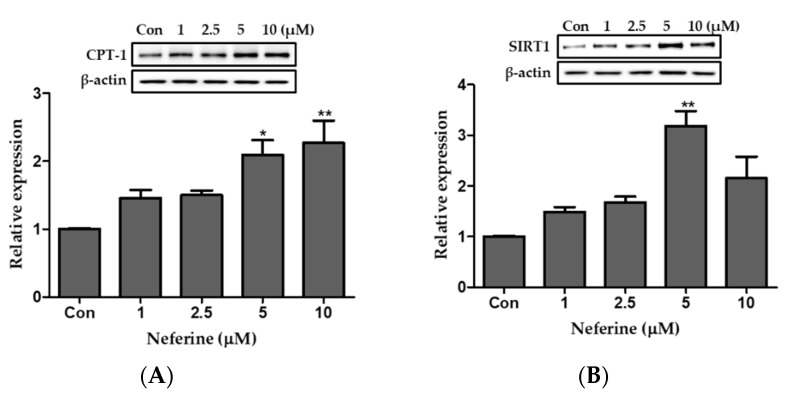
Effect of neferine on the expression of the protein involved in fatty acid oxidation. Western blotting was carried out to analyze the protein expression of (**A**) CPT-1 and (**B**) SIRT1 following normalization to β-actin. * *p* < 0.05, ** *p* < 0.01, and *** *p* < 0.001 vs. Con. All data are presented as mean ± SD, and experiments were performed at least thrice. The positive control (Con) was differentiated 3T3-L1 cells treated with adipocyte differentiation cocktail.

We differentiated 3T3-L1 cells into mature adipocytes and prepared three identical immunoblots to study the effect of neferine on fatty acid oxidation. Relative CPT-1 protein expression was significantly upregulated following neferine treatment in a dose-dependent manner (Figure 5A). Neferine increased the expression of sirtuin 1 (SIRT1) at concentrations up to 5 μM (Figure 5B).

### 3.5. Effect of Neferine on the AMPK Pathway of 3T3-L1 Adipocytes

**Figure 6:**
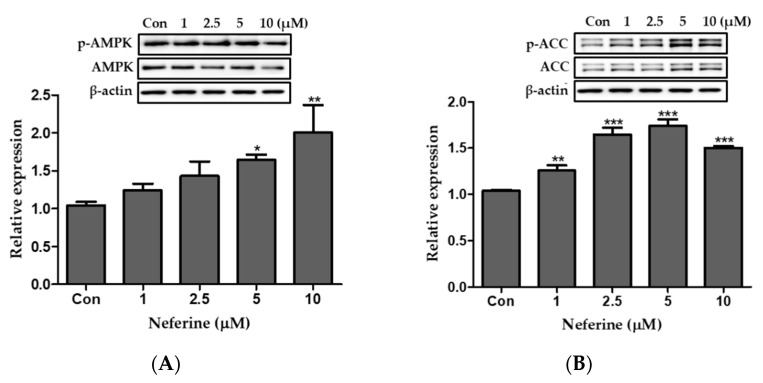
Effects of neferine on AMPK and ACC during the differentiation of 3T3-L1 adipocytes. Ratios of relative expression levels of (**A**) *p*-AMPK/AMPK and (**B**) *p*-ACC/ACC are presented. * *p* < 0.05, ** *p* < 0.01, and *** *p* < 0.001 vs. Con. All data are presented as mean ± SD, and experiments were performed at least thrice. The positive control (Con) was differentiated 3T3-L1 cells treated with adipocyte differentiation cocktail.

We studied the effect of neferine on the AMPK pathway of 3T3-L1 adipocytes using western blot analysis. The ratio of *p*-AMPK/AMPK increased following neferine treatment in a dose-dependent manner, as indicated in Figure 6A. Phosphorylation of ACC also significantly increased following treatment with neferine at concentrations up to 5 μM (Figure 6B). Thus, neferine activated the signaling mediated by AMPK.

### 3.6. Effect of Neferine on the Adipogenesis of Primary White Adipocytes

**Figure 7:**
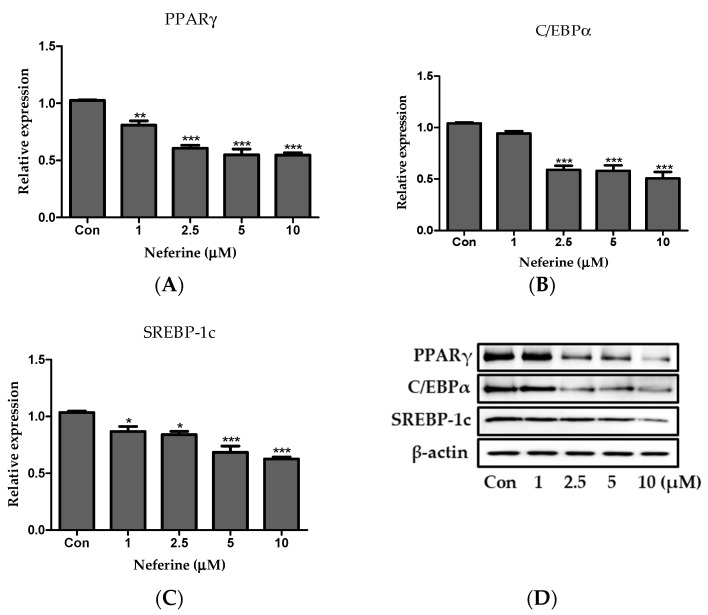
Effects of neferine on adipogenesis of primary white adipocytes. Protein expression levels of (**A**) PPARγ, (**B**) C/EBPα, and (**C**) SREBP-1c were investigated. (**D**) Detected bands of adipogenic factors. Results are expressed following normalization of values to β-actin level. * *p* < 0.05, ** *p* < 0.01, and *** *p* < 0.001 vs. Con. All data are presented as mean ± SD, and experiments were performed at least thrice. The positive control (Con) was differentiated primary white adipocytes treated with differentiation cocktail.

Primary white adipocytes were isolated from the subcutaneous and epididymal adipose tissues of C57BL/6 mice to examine the effect of neferine on adipogenic factors. Primary white adipocytes treated with neferine showed a consdierbale decrease in the relative protein expression of PPARγ, C/EBPα, and SREBP-1c in a neferine concentration-dependent manner, consistent with the results observed with 3T3-L1 adipocytes (Figure 7A–D).

### 3.7. Effect of Neferine on the AMPK Pathway of Primary White Adipocytes

**Figure 8:**
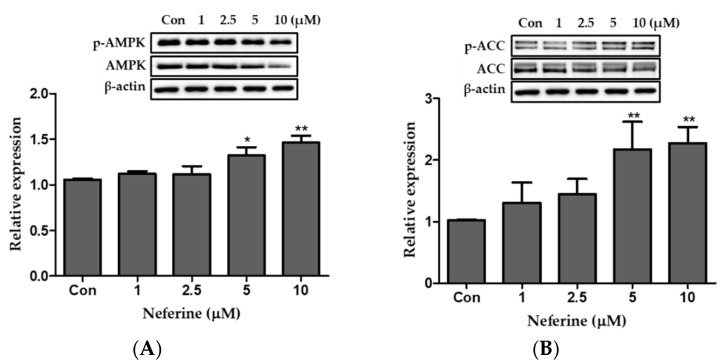
Effects of neferine on AMPK and ACC in primary white adipocytes. Western blot analysis was performed to evaluate the ratio of the relative protein expression levels of (**A**) *p*-AMPK/AMPK and (**B**) *p*-ACC/ACC. * *p* < 0.05 and ** *p* < 0.01 vs. Con. All data are presented as mean ± SD, and experiments were performed at least thrice. The positive control (Con) was differentiated primary white adipocytes treated with differentiation cocktail.

To investigate the effect of neferine on the AMPK pathway of primary white adipocytes, three identical western blots were prepared. AMPK and ACC expression was dose-dependently upregulated by neferine treatment (Figure 8A,B). Together, these data demonstrated that neferine activates the AMPK signaling pathway in primary white adipocytes.

### 3.8. Effect of Neferine on the AMPK Pathway of 3T3-L1 Adipocytes

**Figure 9:**
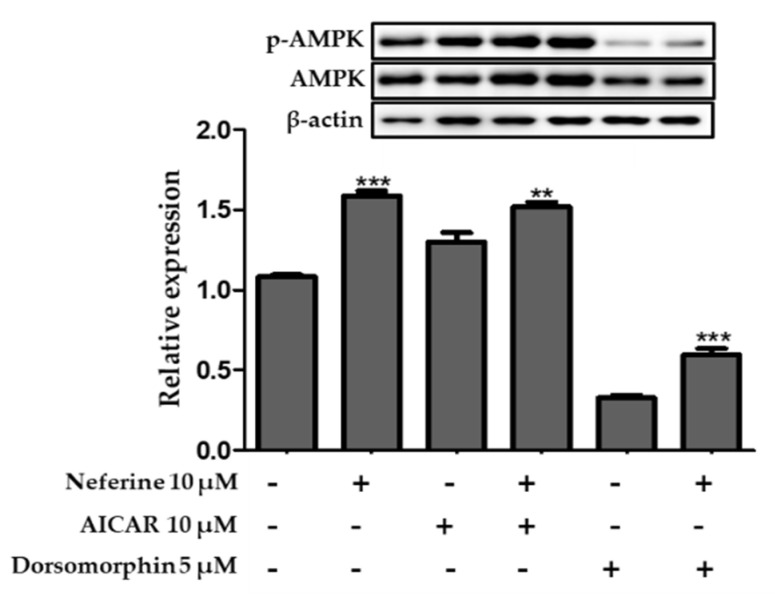
Effect of neferine in 3T3-L1 adipocytes treated with an inhibitor (dorsomorphin) and activator (5-aminoimidazole-4-carboxamide ribonucleotide (AICAR)) of AMPK. ** *p* < 0.01 and *** *p* < 0.001 vs. without Neferine. The ratio of *p*-AMPK/AMPK was analyzed using immunoblotting. All data are presented as mean ± SD, and experiments were performed at least thrice.

To confirm whether AMPK activation was involved in mediating the anti-adipogenic effects of neferine, 3T3-L1 cells were treated with an AMPK inhibitor dorsomorphin (5 µM) and AMPK activator AICAR (10 µM). As described in Figure 9, the protein expression level of AMPK increased following AICAR treatment but reduced after dorsomorphin treatment. Neferine treatment significantly upregulated AMPK expression as compared to control treatment. These data demonstrate that the AMPK pathway plays an important role in mediating the anti-adipogenic effects of neferine in 3T3-L1 adipocytes.

## 4. Discussion

Obesity, a growing pandemic, is associated with various metabolic disorders. Numerous researches have been directed to ameliorate obesity and related complications [31,32]. As most anti-obesity drugs exert side-effects [33], plant-based phytochemicals are gaining attention. In general, lipid droplet accumulation and preadipocytes differentiation into mature adipocytes are regarded as the hallmark events in obesity [32,34]. The present study suggests that neferine prominently reduces lipid accumulation and differentiation of 3T3-L1 adipocytes and primary white adipocytes by regulating adipogenic transcriptional factors and AMPK pathway.

The differentiation of 3T3-L1 preadipocytes is mainly mediated by critical nuclear transcription factors, PPARγ and C/EBPα [35]. C/EBPβ and C/EBPδ are stimulated in the process of differentiation, thereby inducing the expression of PPARγ and C/EBPα [36]. Even without hormones, differentiation of preadipocytes is induced by the expression of PPARγ. Thus, PPARγ may be a core factor involved in adipogenesis [37]. C/EBPα is expressed along with PPARγ after the end of growth during the adipogenic stage [38] and associated with lipid metabolism [39]. PPARγ and C/EBPα control the positive feedback loop to mediate adipogenesis [40]. SREBP-1c is also a vital regulator involved in adipocyte differentiation and lipid metabolism and participates in lipogenesis [24,41]. In this study, neferine downregulated the expression of adipogenic/lipogenic mRNAs and proteins, including PPARγ, C/EBPα, and SREBP-1c, in 3T3-L1 adipocytes and primary white adipocytes. In addition, Oil Red O staining demonstrated the neferine-mediated inhibition of intracellular lipid accumulation.

AMPK plays a key role in mitochondrial energy homeostasis and regulates lipid and fatty acid metabolism [42,43]. AMPK is known to exert beneficial effects in many tissues, including the adipose tissue, and activation of AMPK is known to suppress adipogenesis by reducing the expression of adipogenic factors [44]. ACC, a major regulator of mitochondrial fatty acid oxidation, is phosphorylated upon AMPK activation [45]. In the present study, the cells treated with neferine showed upregulated AMPK expression and ACC phosphorylation.

SIRT1 is an NAD-dependent protein that separates acetyl groups from various proteins [46]. SIRT1, Like AMPK, is involved in cellular processes such as energy and lipid metabolism and mitochondrial biogenesis, and controls adipokines in the adipose tissue [47,48]. CPT-1 is associated with fatty acid metabolism and imports the acyl group of long-chain fatty acids to mitochondria to generate acyl carnitines [49,50]. Further, the activation of AMPK and SIRT1 induces β-oxidation by stimulating CPT-1 expression [51]. Here, we found that the protein expression levels of SIRT1 and CPT-1 were increased in 3T3-L1 adipocytes following neferine treatment.

As previously stated, neferine upregulated *p*-AMPK/AMPK and *p*-ACC/ACC ratios. Moreover, the AMPK activity of neferine-treated 3T3-L1 cells was promoted by AICAR, an AMPK agonist, and suppressed by the AMPK antagonist dorsomorphin. We showed that the anti-adipogenic effect of neferine was related to AMPK-mediated regulation.

Taken together, our study demonstrates that neferine prominently inhibits the accumulation of intracellular lipid and differentiation of 3T3-L1 and primary white adipocytes into mature adipocytes at moderate concentrations through the AMPK signaling pathway. Overall, we verify that neferine may exhibit potential therapeutic properties for obesity management. Further investigation is warranted to demonstrate the underlying mechanism and substantiate the safety and value of neferine.

## Figures and Tables

**Table 1:** Primer sets for real-time quantitative polymerase chain reaction.

Gene	Forward (5′–3′)	Reverse (5′–3′)
PPARγ	TTTTCAAGGGTGCCAGTTTC	AATCCTTGGCCCTCTGAGAT
C/EBPα	TTACAACAGGCCAGGTTTCC	GGCTGGCGACATACAGTACA
SREBP-1	TGTTGGCATCCTGCTATCTG	AGGGAAAGCTTTGGGGTCTA
β-actin	CTGTCCCTGTATGCCTCTG	ATGTCACGCACGATTTCC

PPARγ: peroxisome proliferator-activated receptor gamma, C/EBPα: CCAAT/enhancer-binding protein alpha, SREBP-1: Sterol regulatory element-binding transcription factor-1.
